# TGF-β-induced interleukin-6 participates in transdifferentiation of human Tenon’s fibroblasts to myofibroblasts

**Published:** 2009-10-21

**Authors:** Gong Je Seong, Samin Hong, Sun-Ah Jung, Jung-Jin Lee, Eunhae Lim, Sung-Joo Kim, Joon H. Lee

**Affiliations:** 1 Institute of Vision Research, Department of Ophthalmology, Yonsei University College of Medicine, Seoul, Korea; 2 Myunggok Eye Research Institute at Kim’s Eye Hospital, Konyang University College of Medicine, Seoul, Korea

## Abstract

**Purpose:**

To gain a better understanding of the roles of interleukins (ILs) in subconjunctival fibrosis, we investigated their expression in transforming growth factor-β1 (TGF-β1)-stimulated Tenon’s fibroblasts and examined their association with the transdifferentiation of fibroblasts to myofibroblasts.

**Methods:**

After primary culture, fibroblasts derived from human Tenon’s capsule were exposed to TGF-β1. The expression of α-smooth muscle actin (α-SMA) protein was assessed by western immunoblots and immunofluorescence. The mRNA levels of various ILs were also evaluated by multiplex reverse transcription (RT)-PCR. Using the small interfering RNAs (siRNAs) specific for *IL-6* and *IL-11* and the promoter deletion assay, the contributions of *IL-6* and *IL-11* to TGF-β1-induced induction of α-SMA were determined.

**Results:**

In human Tenon’s fibroblasts, TGF-β1 stimulated the expression of α-SMA protein determined by western blot analysis and also increased the mRNA levels of *IL-6* and *IL-11* determined by multiplex RT-PCR. On the western immunoblots and immunofluorescence, the increased expression of α-SMA was attenuated only by the siRNAs specific for *IL-6* but not by the siRNAs specific for *IL-11*. When the activator protein-1 binding sites of the *IL-6* promoter region were deleted, the stimulation effects of TGF-β1 decreased.

**Conclusions:**

Our data show that autocrine IL-6 may participate in the TGF-β1-induced transdifferentiation of human Tenon’s fibroblasts to myofibroblasts, which is known to be an essential step for subconjunctival fibrosis.

## Introduction

Subconjunctival fibrosis is an essential wound-healing process of the ocular surface, but if excessive it can result in ocular morbidity, as seen in patients with oculocutaneous disorders, such as ocular cicatricial pemphigoid, and patients who have undergone glaucoma-filtering surgery [1-6]. Even though transforming growth factor-β (TGF-β) is known to play a crucial role in this fibrosis [7-9], detailed mechanisms of how it functions have not yet been elucidated. Several recently published research papers that demonstrated antifibrotic effects of anti-TGF-β molecules have re-stimulated interest in TGF-β-mediated fibrosis [10-14].

In the present study, we were interested in investigating the relationship between inflammation and fibrosis in human Tenon’s fibroblasts. In lung and heart, certain types of inflammation recruit and stimulate fibroblasts in a TGF-β-dependent manner [15-18]. These activated fibroblasts then transdifferentiate to myofibroblasts that produce extracellular matrix (ECM); these contractile cells ultimately cause extensive fibrosis. In this study we investigated which of the proinflammatory cytokines of the interleukin (IL) family are stimulated by TGF-β1, and we monitored changes in α-smooth muscle actin (α-SMA), a phenotypic hallmark of myofobroblasts [19], to investigate the effect of the TGF-β1-stimulated ILs on the transdifferentiation of fibroblasts to myofibroblasts. The effects of blocking these ILs with small interfering RNA (siRNA) were also investigated.

## Methods

### Cell culture

After obtaining approval from the Institutional Review Board of our institution, 6 human Tenon’s capsule specimens were excised during strabismus surgeries in compliance with the provisions of the Declaration of Helsinki. A total of six participants who have no other disease except strabismus and no previous ocular surgery/trauma history were included. Written informed consent was obtained before operative excision. Briefly, 5x5-mm sections of Tenon’s capsule were collected, minced, and placed in a 35-mm culture dish containing Dulbecco’s modified Eagle’s medium (DMEM; Invitrogen Co., Carlsbad, CA) supplemented with 10% fetal calf serum (Invitrogen), and 50 U/ml penicillin and 50 μg/ml streptomycin (Invitrogen). Cells were allowed to migrate from the explanted tissue and were then incubated at 37 °C and 5% CO_2_. Cells between the third and fifth passages were used for this study. Cultures were allowed to reach about 80% confluence. Depending on the experiments, fibroblasts were treated with various concentrations of TGF-β1 (R&D System, Minneapolis, MN) after 24 h of serum starvation in serum-free DMEM.

### Western immunoblot analysis

Whole cellular proteins were isolated from primary cultured fibroblasts of human Tenon’s capsules, as described previously [20]. Briefly, total cell lysates were obtained by using lysis buffer (25 mM HEPES [pH 7.5], 0.3 M NaCl, 1.5 mM MgCl_2_, 0.2 mM EDTA, 0.05% Triton X-100, 0.5 mM dithiothreitol [DTT], and 0.4 mM phenylmethylsulfonyl fluoride [PMSF]; Sigma-Aldrich, Co., St. Louis, MO), 2 μg/ml leupeptin (Sigma-Aldrich), and 2 μg/ml aprotinin (Sigma-Aldrich). Equal amounts of protein (20 μg) were subjected to sodium dodecyl sulfate polyacrylamide gel electrophoresis and transferred to nitrocellulose membranes. Membranes were probed with primary antibodies against human α-SMA (diluted 1:500; Dako Corporation, Carpinteria, CA) and β-actin (diluted 1:5,000; Santa Cruz Biotechnology, Inc., Santa Cruz, CA). Immunoreactive bands were detected with horseradish peroxidase-conjugated secondary antibodies (diluted 1:5,000; Invitrogen) and visualized by enhanced chemiluminescence detection reagents on autoradiograph films.

### Multiplex reverse transcription-PCR

Total RNA was extracted from fibroblasts and converted into complementary (c)DNA by a first-strand synthesis system (SuperScript III; Invitrogen). Subsequently, the cDNA was used as a template for multiplex reverse transcription (RT)-PCR assays (MegaXpression; Seegene, Inc., Seoul, Korea) [21,22]. The following IL gene segments were amplified: *IL-1α*, *IL-1β*, *IL-2*, *IL-3*, *IL-4*, *IL-5*, *IL-6*, *IL-7*, *IL-8*, *IL-9*, *IL-10*, *IL-11*, *IL-12A*, *IL-12B*, *IL-13*, *IL-15*, *IL-16*, *IL-18*, and IL-1 receptor antagonist (*IL-1RN*). In addition, gene segments of *β-actin*, β2-microblobulin (*β2M*), glyceraldehyde-3-phosphate dehydrogenase (*GAPDH*), succinate dehydrogenase complex subunit A (*SDHA*), and ribosomal protein L13a (*RPL13a*) were amplified as housekeeping genes.

### RNA interference assay

siRNA molecules targeting *IL-6* and *IL-11* mRNA were purchased from Ambion, Inc. (Austin, TX). The RNA duplex against *IL-6* had the sequence 5'-GGA CAU GAC AAC UCA UCU CTT-3' (sense) and 5'-GAG AUG AGU UGU CAU GUC CTG-3' (antisense); and the RNA duplex against *IL-11* had the sequence 5'-GCA ACA UGG UGC AUC UGU GTT-3' (sense) and 5'-CAC AGA UGC ACC AUG UUG CTT-3' (antisense). siRNAs were delivered into cells according to the manufacturer’s instructions. Briefly, the diluted transfection reagent (siPORT Amine; Ambion) was mixed with the diluted siRNA to allow the formation of transfection complexes. This mixture with a final RNA concentration of 20 nM was then dispensed onto the cells and incubated for 24 h.

### Immunofluorescence analysis

To carry out immunostaining, fibroblasts were fixed in 4% paraformaldehyde for 5 min and permeabilized in 0.2% Triton X-100 for 5 min at room temperature. Fibroblasts were then incubated with the primary antibody against human α-SMA (diluted 1:50) overnight at 4 °C and probed with a fluoroscein isothiocyanate-conjugated secondary antibody (diluted 1:100) for 2 h at room temperature in the dark. Subsequently, they were washed and viewed by immunofluorescence microscopy (IX71; Olympus, Tokyo, Japan).

### Cell transfection and luciferase assay

Three recombinant plasmids, in which different fragments of the activator protein-1 (AP-1)-binding site of the human *IL-6* promoter were deleted (p1168hu.IL6P-luc+, wild type; p1168h.IL6m3AP1-luc+, 3'-terminal deletion; and p1168h.IL6m5AP1-luc+, 5'-terminal deletion), were used as reporter constructs. These plasmids were obtained from the Belgian Coordinated Collections of Microorganism (BCCM/LMBP) Plasmid Collection (Zwijnaarde, Belgium) and have been described previously [23,24].

Cells were transfected with an equal amount of reporter plasmid using Lipofectamine 2000 (Invitrogen). The luciferase activities were determined using a luciferase assay system (Promega, Co., Madison, WI), following the manufacturer’s instruction. Briefly, the 96-well plate containing 20 μl of cell lysate per well was placed into the luminometer with injector. The injector added 100 μl of luciferase assay reagent per well, then the well was read immediately. The plate was advanced to the next well for a repeat of the inject-then-read process. The typical delay time was 2 s and the typical read time was 10 s.

### Data analysis

Densitometric data are expressed as means ± standard deviation (SD). Data were compared with Kruskal-Wallis one-way analysis of variance and Mann-Whitney *U* test using the SPSS program for Windows, version 12.0.1 (SPSS Inc., Chicago, IL). A p value less than 0.05 was considered statistically significant.

## Results

### Transforming growth factor-β1 induces transdifferentiation of fibroblasts to myofibroblasts

**Figure 1:**
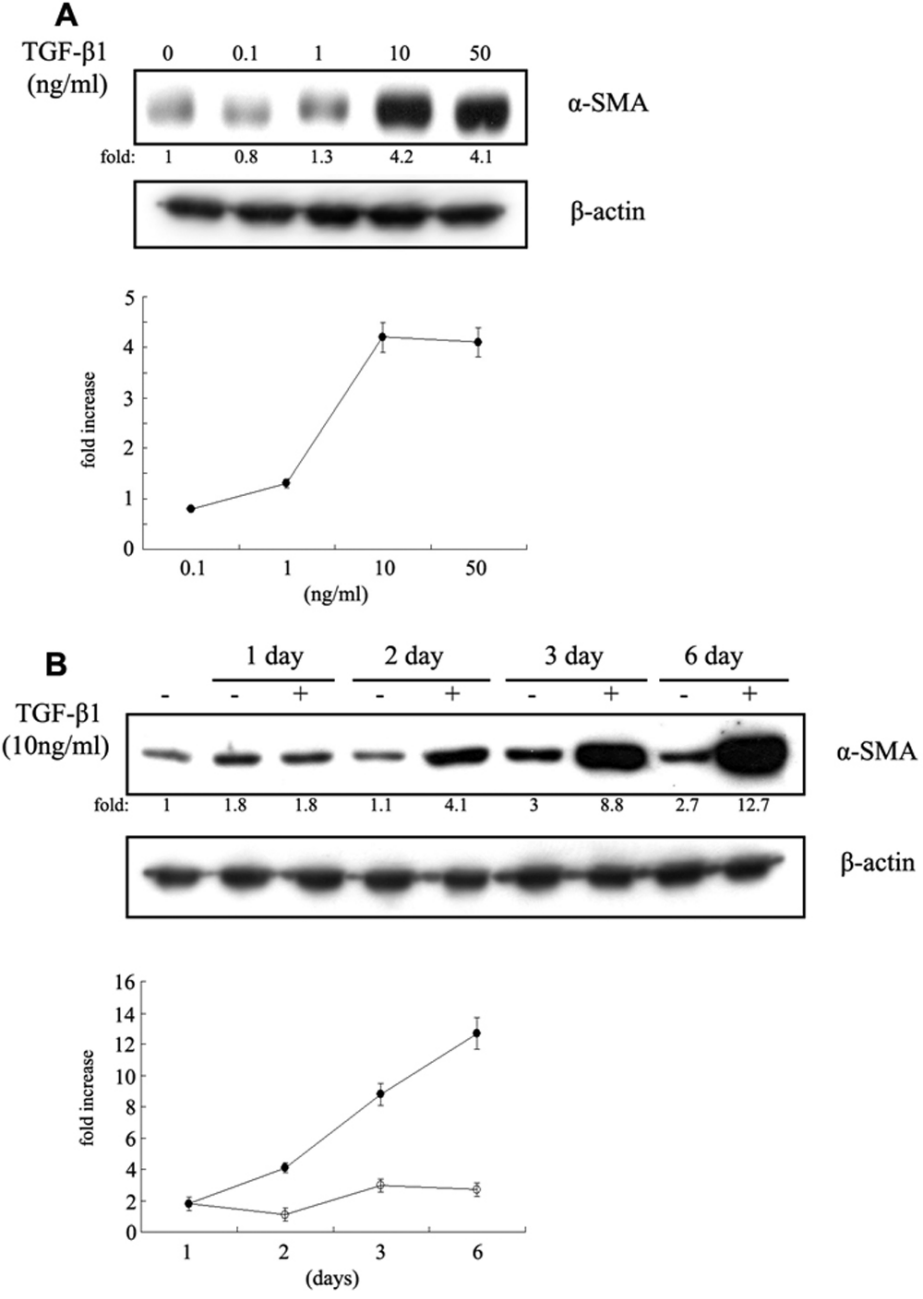
Representative western blot bands for α-smooth muscle actin. **A**: Dose-response effects: fibroblasts were exposed to various concentrations of  transforming growth factor (TGF)-β1 for 72 h. **B**: Time-response effects: fibroblasts were incubated with or without 10 ng/ml of TGF-β1 for up to 6 days. β-actin was used as an internal control. Densitimetric data represent the mean±SD of results from three independent experiments.

To evaluate the effect of TGF-β1 on the expression of α-SMA, we measured α-SMA protein levels in human Tenon’s fibroblasts stimulated with different concentrations of TGF-β1 for different durations. Exposure of fibroblasts to TGF-β1 resulted in a dose- and time-dependent increase in expression of α-SMA (Figure 1). TGF-β1 treatment (10 ng/ml) for 72 h resulted in a significant increase (p<0.001) in α-SMA expression. Because the presence of α-SMA is a phenotypic hallmark of myofobroblasts, this finding indicates that TGF-β1 induced the transdifferentiation of fibroblasts to myofibroblasts.

### Upregulation of *IL-6* and *IL-11* by transforming growth factor-β1 treatment

**Figure 2:**
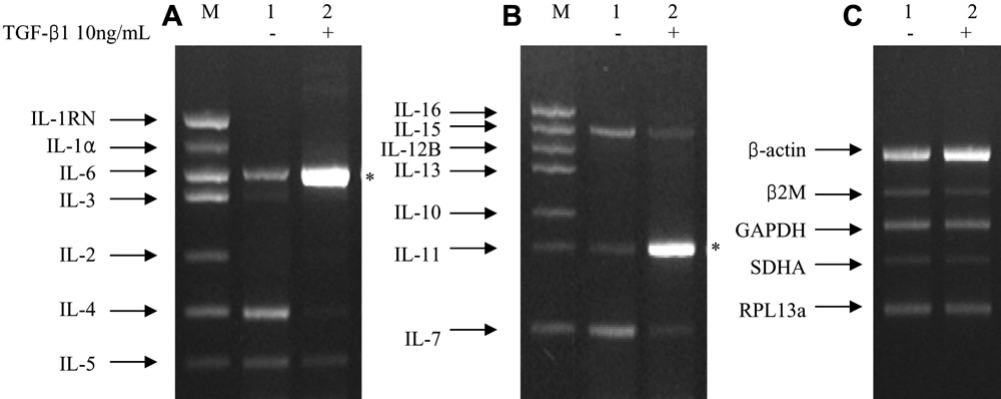
Multiplex reverse transcription PCR. Multiplex reverse transcription PCR sets for interleukins (ILs; **A**, **B**) and housekeeping genes (**C**). For each set of data, lane 1 represents the control group with no transforming growth factor (TGF)-β1 treatment and lane 2 represents the study group with 10 ng/ml TGF-β1 treatment for 72 h. Lane M contains positive markers. *GAPDH*, glyceraldehyde-3-phosphate dehydrogenase;* IL-1RN*, interleukin-1 receptor antagonist; *RPL13a*;* SDHA*, succinate dehydrogenase complex subunit A; *β2M*, β2-microblobulin. Two asterisks refer to a significant increase (p<0.001) in expression of *IL-6* and *IL-11* in the presence of TGF-β1.

To assess the effect of TGF-β1 on the transcription of ILs, the IL mRNA levels in TGF-β1-treated human Tenon’s fibroblasts were evaluated using multiplex RT-PCR. After fibroblasts were incubated with 10 ng/ml TGF-β1 for 72 h, multiplex RT-PCR was performed. For the first set of genes (*IL-1RN*, *IL-1α*, *IL-2*, *IL-3*, *IL-4*, *IL-5*, and *IL-6*), only *IL-6* mRNA was increased significantly (p<0.001; Figure 2A); for the second set of genes (*IL-7*, *IL-10*, *IL-11*, *IL-12B*, *IL-13*, *IL-15*, and *IL-16*), only *IL-11* mRNA was increased significantly (p<0.001; Figure 2B). The levels of *IL-1β*, *IL-8*, *IL-9*, *IL-12A*, and *IL-18* were not changed (data not shown) and the expression of *β-actin*, *β2M*, *GAPDH*, *SDHA*, and *RPL13a* was not influenced by TGF-β1 (Figure 2C). These data demonstrate that TGF-β1 induces the transcription of *IL-6* and *IL-11* in human Tenon’s fibroblasts.

### Transforming growth factor-β1-induced expression of α-smooth muscle actin is reduced by *IL-6*-specific small interfering RNAs

**Figure 3:**
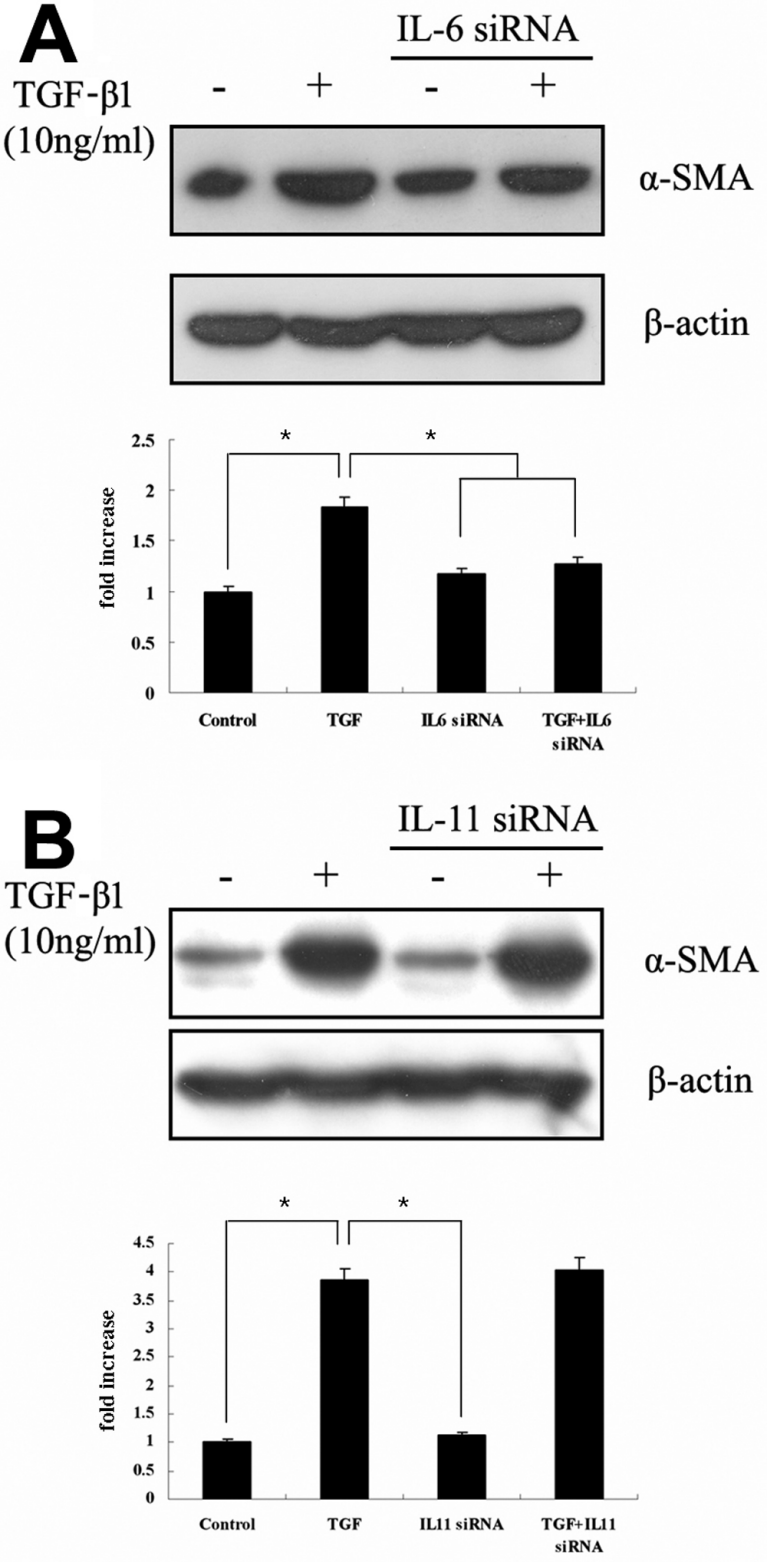
RNA interference assay for *IL-6* and *IL-11*. After administering small interfering RNAs targeting *IL-6* (**A**) and *IL-11* (**B**), fibroblasts were exposed to 10 ng/ml transforming growth factor (TGF)-β1 for 72 h. Then, the expression of α-smooth muscle actin (α-SMA) was evaluated using western blots. Densitimetric data represent the mean±SD of results from three independent experiments. The asterisks refer to a significant difference compared to TGF-β treatment only (p<0.001).

To more directly address the possible involvement of IL-6 and IL-11 in TGF-β1-induced myofibroblast transdifferentiation, we evaluated the effects of *IL-6* and *IL-11* knockdown using specific siRNAs. Since the accumulation of ECM proteins is an indicator of myofibroblast transdifferentiation, we studied the effect of *IL-6*- and *IL-11*-specific siRNAs on TGF-β1-induced expression of the ECM component α-SMA. Treatment with *IL-6*-specific siRNAs strongly inhibited the TGF-β1-induced α-SMA expression (Figure 3A), whereas *IL-11*-specific siRNAs had little effect (Figure 3B).

**Figure 4:**
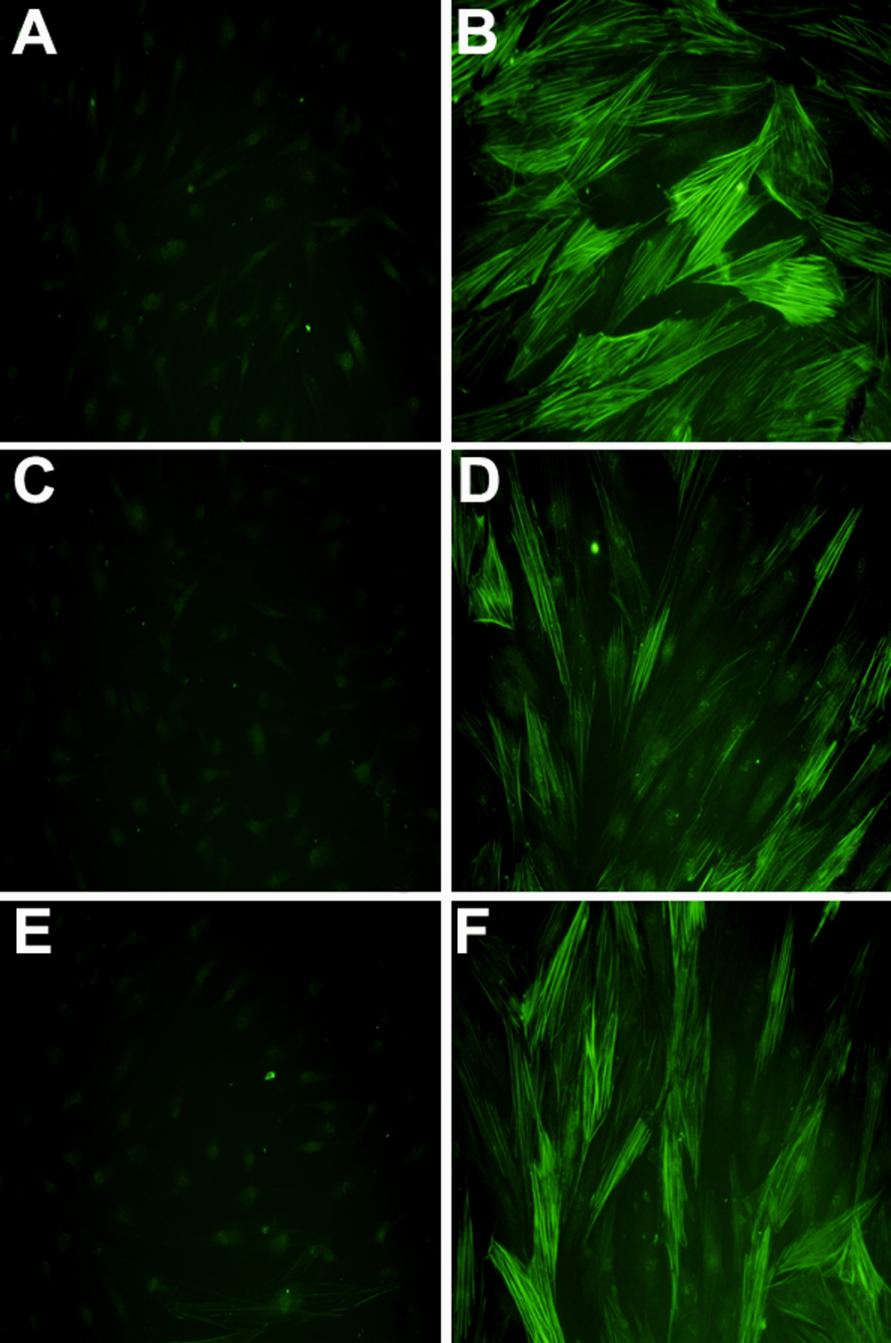
Immunofluorescent analysis of the expression of α-smooth muscle actin. **A**: No treatment control, **B**: 10 ng/ml transforming growth factor (TGF)-β1 for 72 h, **C**: *IL-6*-specific small interfering (si)RNA, **D**: *IL-6*-specific siRNA plus 10 ng/ml TGF-β1 for 72 h, **E**: *IL-11*-specific siRNA, and **F**: *IL-11*-specific siRNA plus 10 ng/ml TGF-β1 for 72 h. The secondary antibody was conjugated with fluoroscein isothiocyanate, resulting in green fluorescence. Total magnification was 400×.

To confirm this finding, we assessed the effects of *IL-6*- and *IL-11*-specific siRNAs on TGF-β1-induced α-SMA upregulation by immunofluorescent staining and obtained similar results with western immunoblot analysis (Figure 4). Compared to untreated control fibroblasts, TGF-β1-stimulated fibroblasts showed a strong cytoplasmic α-SMA signal with numerous intracellular stress fibers (Figure 4B). Fibroblasts transfected with *IL-6*-specific siRNAs showed a significant reduction in the effect of TGF-β1 on α-SMA expression (Figure 4D); but cytoplasmic staining for α-SMA was little diminished in the presence of *IL-11*-specific siRNAs (Figure 4F). These data demonstrate that knockdown of *IL-6* reduces the ability of TGF-β1 to induce α-SMA expression.

### Activator protein-1 activity at the *IL-6* promoter in the presence of transforming growth factor-β1

**Figure 5:**
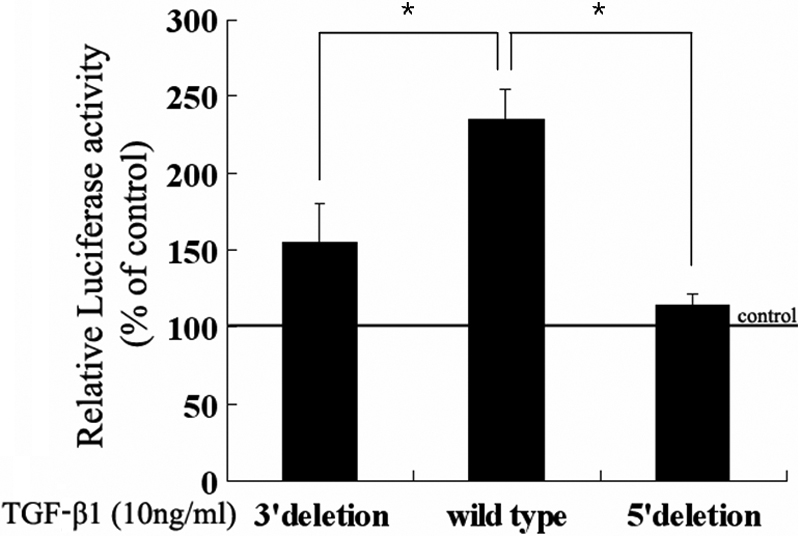
Promoter deletion assay for interleukin-6 (*IL-6*). Three recombinant plasmids, in which different fragments of the activator protein-1 (AP-1)-binding site on the human *IL-6* promoter were deleted, were used. After transfection with each plasmid, fibroblasts were incubated with 10 ng/ml transforming growth factor (TGF)-β1 for 72 h. The luciferase activity was then determined and expressed as the percent of the no-TGF-β1 treatment control. Data represent the mean±SD of results from three independent experiments. The asterisks refer to a significant difference (p<0.001) compared to the wild type.

To determine whether TGF-β1-mediated myofibroblast transdifferentiation might be related to AP-1-binding site-dependent effects on *IL-6* transcription, we used wild-type and mutant *IL-6* promoter-reporter constructs to analyze *IL-6* promoter activity (Figure 5). TGF-β1 increased the promoter activity of the wild-type-reported plasmid about 2.5-fold above the no-treatment control (p<0.001). However when the 3' or 5' end of the AP-1-binding site of the *IL-6* promoter was deleted, the increased promoter activity by TGF-β1 was significantly attenuated (p<0.001). Our results indicate that the effect of TGF-β1 on IL-6 expression is dependent on the AP-1-binding site of the *IL-6* promoter region and suggest that TGF-β1-induced myofibroblast transdifferentiation is dependent, at least in part, on TGF-β1-induced IL-6 expression.

## Discussion

Transdifferentiation of fibroblasts to myofibroblasts is at the core of the fibrotic process. Although the fibrosis caused by these activated fibroblasts is essential for natural wound healing, it can also result in excessive scarring, which is one of the most common causes of surgical failure after various ocular surgeries, including glaucoma-filtering procedures [1-6]. Although adjunctive antimetabolites can suppress the proliferation of fibroblasts and reduce subconjunctival fibrosis, the use of these compounds may result in serious complications; thus a new treatment strategy to prevent excessive fibrosis is required.

In various organs, including the lung and heart, inflammation is known to result in the development of fibrosis; TGF-β seems to be at the center of this process [15-18]. At the ocular surface, TGF-β plays a key role as a potent fibrogenic cytokine [25-29]. It is thought to stimulate the chemotaxis and transdifferentiation of fibroblasts, resulting in the overproduction of collagen, fibronectin, and other ECM components. Furthermore, TGF-β reduces the degradation of synthesized ECM through suppression of the activity of matrix metalloproteases and the activation of protease inhibitors.

IL-6 is essentially a chemoattractant and stimulator of lymphocytes [30-33]. It also functions as a pleiotropic mediator in the acute-phase response and stimulates the differentiation and proliferation of various target cells [33]. IL-6 exerts its effects through the Janus kinase/signal transducer and activator of transcription (JAK/STAT) pathway [34]. Because the IL-6 receptor does not have intrinsic tyrosine kinase activity, when IL-6 binds to the extracellular ligand-binding domain of its receptor, members of the JAK family are activated and phosphorylate the tyrosine residues of the IL-6 receptor, which then act as docking sites for the SH2 domains of STATs. IL-6-activated T lymphocytes then secrete TGF-β and trigger the fibrotic cascade.

Consistent with a previous report that IL-6 participates directly in the transdifferentiation of Tenon’s capsule fibroblasts to myofibroblasts [35], in the present study we verified the presence of autocrine IL-6 from activated Tenon’s capsule fibroblasts.

We found that the transcription of *IL-6* and *IL-11* was increased by TGF-β1 stimulation in human Tenon’s fibroblasts, and that this was accompanied by the upregulated expression of α-SMA. When *IL-6*-specific siRNAs were used, the TGF-β1-stimulated increase in expression of α-SMA was blocked, but this did not occur when *IL-11*-specific siRNAs were used. Our results indicate that autocrine IL-6 produced as a result of TGF-β-stimulation of human Tenon’s fibroblasts stimulates the transdifferentiation of these fibroblasts to myofibroblasts, which is thought to be essential for subconjunctival fibrosis. Modulation of autocrine IL-6 production might be useful as a novel therapeutic strategy for controlling postoperative subconjunctival fibrosis.
